# Supplementary material for: From sequence to enzyme mechanism using multi-label machine learning
Source: BMC Bioinformatics. 2014 May 19;15:150. doi: 10.1186/1471-2105-15-150 (PMC4229970; doi:10.1186/1471-2105-15-150)
Supplement: Additional file 2 — Java code of ml2db. Additional file ml2db_code.tar.gz contains the Java source code to run the multi-label machine learning experiments and save the results to database. The code’s Javadoc is included. [file 1471-2105-15-150-S2.zip › additional file 2/ml2db/ml2db/doc/index-files/index-4.html]

D-Index


JavaScript is disabled on your browser.


- Overview
- Package
- Class
- Use
- Tree
- Deprecated
- Index
- Help

- Prev Letter
- Next Letter

- Frames
- No Frames

- All Classes

A B C D E F G H I J K L M N O P Q R S T U V W X 


## D

DATA\_LOCAL\_DB - Static variable in class main.ml2db.ProjectParameters


DATA\_PROPERTIES\_FILE\_PATH\_PROP - Static variable in class main.ml2db.weka.ArffPropsFromFile
:   the path to the properties file itself, from which the other properties
    were read

DATA\_REMOTE\_DB - Static variable in class main.ml2db.ProjectParameters


DatabaseTest - Class in test.ml2db.dataharness
:   Check the test database

DatabaseTest() - Constructor for class test.ml2db.dataharness.DatabaseTest


dataMapsAreCorrect(InstAttValueMap, InstAttValueMap) - Method in class main.ml2db.weka.DataSetChecker
:   public boolean initialisationIsCorrect() { return
    m\_initialisationIsCorrect; }
    public void setInitialisation(boolean isCorrect) {
    m\_initialisationIsCorrect = isCorrect;
    }

DataOne - Class in test.ml2db.dataharness
:   Class

DataOne() - Constructor for class test.ml2db.dataharness.DataOne


DATASET\_NAME - Static variable in class test.ml2db.dataharness.DataOne


DATASET\_NAME - Static variable in class test.ml2db.dataharness.DataTwo


DATASET\_NAME\_PROP - Static variable in class main.ml2db.weka.ArffProps
:   the experiment short name

DataSetChecker - Class in main.ml2db.weka
:   Checks that all data (or file paths) needed to build the ARFF file are
    available

DataSetChecker(DataSetManager) - Constructor for class main.ml2db.weka.DataSetChecker


DataSetCheckerTest - Class in test.ml2db.weka
:   Class

DataSetCheckerTest() - Constructor for class test.ml2db.weka.DataSetCheckerTest


DataSetDbLoader - Class in main.ml2db.weka
:   Loads data (instances, attribute values, class values) from database using
    the queries in the data Properties.

DataSetDbLoader(DataSetManager) - Constructor for class main.ml2db.weka.DataSetDbLoader


DataSetDbLoaderTest - Class in test.ml2db.weka
:   Class

DataSetDbLoaderTest() - Constructor for class test.ml2db.weka.DataSetDbLoaderTest


DataSetGenerator - Class in main.ml2db.weka
:   Generates and fills the Weka instances.

DataSetGenerator(DataSetManager, InstAttValueMap, InstAttValueMap) - Constructor for class main.ml2db.weka.DataSetGenerator


DataSetGeneratorTest - Class in test.ml2db.weka
:   Class

DataSetGeneratorTest() - Constructor for class test.ml2db.weka.DataSetGeneratorTest


DataSetManager - Class in main.ml2db.weka
:   DataSet generates a collection of Weka instances (rows of the ARFF file),
    their attributes (column names) and their attribute values (cell values) The
    last attribute is the class attribute.

DataSetManager(Arff) - Constructor for class main.ml2db.weka.DataSetManager


DataSetManagerTest - Class in test.ml2db.weka
:   Class

DataSetManagerTest() - Constructor for class test.ml2db.weka.DataSetManagerTest


DataSetWriter - Class in main.ml2db.weka
:   Class

DataSetWriter(DataSetManager) - Constructor for class main.ml2db.weka.DataSetWriter


DataSetWriterTest - Class in test.ml2db.weka
:   Class

DataSetWriterTest() - Constructor for class test.ml2db.weka.DataSetWriterTest


dataStatistics() - Method in class main.ml2db.weka.DataSetWriter


DataTableFourTest - Class in test.ml2db.dataharness


DataTableFourTest() - Constructor for class test.ml2db.dataharness.DataTableFourTest


DataTableOneTest - Class in test.ml2db.dataharness
:   Basic data table containing 3 columns with: instances, attributes, classes.

DataTableOneTest() - Constructor for class test.ml2db.dataharness.DataTableOneTest


DataTableTest - Class in test.ml2db.dataharness
:   Create a table for test instances (and their attributes and classes)

DataTableTest() - Constructor for class test.ml2db.dataharness.DataTableTest


DataTableThreeTest - Class in test.ml2db.dataharness


DataTableThreeTest() - Constructor for class test.ml2db.dataharness.DataTableThreeTest


DataTableTwoTest - Class in test.ml2db.dataharness


DataTableTwoTest() - Constructor for class test.ml2db.dataharness.DataTableTwoTest


DataTwo - Class in test.ml2db.dataharness
:   Class

DataTwo() - Constructor for class test.ml2db.dataharness.DataTwo


DbManagerForTest - Class in test.ml2db.alltests


DbManagerForTest() - Constructor for class test.ml2db.alltests.DbManagerForTest


DbManagers - Class in main.ml2db
:   Stores a database connection to the databases containing: 1.

DbManagers(DbManager, DbManager, DbManager) - Constructor for class main.ml2db.DbManagers


DbManagers(String, String, String) - Constructor for class main.ml2db.DbManagers


DEBUG - Static variable in class test.ml2db.dataharness.TestProjectParameters


DebuggingObjectOutputStream - Class in main.ml2db


DebuggingObjectOutputStream() - Constructor for class main.ml2db.DebuggingObjectOutputStream


deleteInstance(String) - Method in class main.ml2db.mulan.mulanarff.IdMultiLabelInstances
:   Remove an instance

density() - Method in class main.ml2db.mulan.mulanarff.Statistics
:   returns the label density of the dataset

deriveXmlPath() - Method in class main.ml2db.mulan.mulanarff.MulanArff


deriveXmlPath() - Method in class main.ml2db.weka.ArffProps


DESCRIPTION\_PROP - Static variable in class main.ml2db.weka.ArffProps


deserialize(String) - Static method in class main.ml2db.mulan.learn.MulanSerializer
:   Get trained learner from file

deserialize(String) - Static method in class main.ml2db.SerializerHelper
:   Get populated java object from file

DIRECTED\_ARROW - Static variable in class main.ml2db.mulan.mulanarff.NeighboursPrinter


distance(Instance, Instance) - Method in class main.ml2db.weka.JaccardDistance


distance(Instance, Instance, double) - Method in class main.ml2db.weka.JaccardDistance


distance(Instance, Instance, double, PerformanceStats) - Method in class main.ml2db.weka.JaccardDistance


distance(Instance, Instance) - Method in class main.ml2db.weka.JaccardDistanceStored


distance(Instance, Instance, double) - Method in class main.ml2db.weka.JaccardDistanceStored


distance(Instance, Instance, double, PerformanceStats) - Method in class main.ml2db.weka.JaccardDistanceStored


distance(Instance, Instance, PerformanceStats) - Method in class main.ml2db.weka.JaccardDistanceStored


distance(Instance, Instance) - Method in class main.ml2db.weka.JaccardEuclideanDistance


DISTANCE\_FUNCTION - Static variable in class main.ml2db.al.database.AlExperimentsTable
:   a string describing the distance function used to calculate the distance
    between two instances

DistanceExperimentDetailsTest - Class in test.ml2db.al.utility.globaldistance
:   Class

DistanceExperimentDetailsTest() - Constructor for class test.ml2db.al.utility.globaldistance.DistanceExperimentDetailsTest


DistancesCalculator - Class in main.ml2db.al.utility.globaldistance
:   Calculates the distance between two instances.

DistancesCalculator(IdMultiLabelInstances, String) - Constructor for class main.ml2db.al.utility.globaldistance.DistancesCalculator
:   The distances are not saved to file.

DistancesCalculator(IdMultiLabelInstances, String, boolean) - Constructor for class main.ml2db.al.utility.globaldistance.DistancesCalculator


DistancesCalculator(IdMultiLabelInstances, String, boolean, NormalizableDistance) - Constructor for class main.ml2db.al.utility.globaldistance.DistancesCalculator


DistancesCalculatorTest - Class in test.ml2db.al.utility.globaldistance
:   Class

DistancesCalculatorTest() - Constructor for class test.ml2db.al.utility.globaldistance.DistancesCalculatorTest


DistancesStorage - Class in main.ml2db.al.utility.globaldistance
:   Stores the distance between couples of instances to save computational time.

DistancesStorage(int) - Constructor for class main.ml2db.al.utility.globaldistance.DistancesStorage


DistancesStorageTest - Class in test.ml2db.al.utility.globaldistance
:   Class

DistancesStorageTest() - Constructor for class test.ml2db.al.utility.globaldistance.DistancesStorageTest


DistancesStoreTest - Class in test.ml2db.al.utility.globaldistance
:   Class

DistancesStoreTest() - Constructor for class test.ml2db.al.utility.globaldistance.DistancesStoreTest


DIVIDER - Static variable in class main.ml2db.al.utility.globaldistance.DistancesStorage
:   the string that separates the two instances

DOT - Static variable in class main.ml2db.graphviz.GraphViz


DOT\_COMMENT\_PREFIX - Static variable in class main.ml2db.graphviz.GraphViz


DOT\_UNDIRECTED\_EDGE - Static variable in class main.ml2db.graphviz.GraphViz


DUMMY\_VALUE - Static variable in class main.ml2db.weka.AttributeFactory


DUMMY\_VALUE - Static variable in class main.ml2db.weka.DataSetManager

A B C D E F G H I J K L M N O P Q R S T U V W X

- Overview
- Package
- Class
- Use
- Tree
- Deprecated
- Index
- Help

- Prev Letter
- Next Letter

- Frames
- No Frames

- All Classes
